# Nutrition and Culinary in the Kitchen Program: a randomized controlled intervention to promote cooking skills and healthy eating in university students – study protocol

**DOI:** 10.1186/s12937-017-0305-y

**Published:** 2017-12-20

**Authors:** Greyce Luci Bernardo, Manuela Mika Jomori, Ana Carolina Fernandes, Claudia Flemming Colussi, Margaret D. Condrasky, Rossana Pacheco da Costa Proença

**Affiliations:** 1 Nutrition Postgraduate Program, Nutrition in Foodservice Research Centre, Federal University of Santa Catarina, Florianópolis, Santa Catarina (SC) Brazil; 2 Nutrition Faculty of Federal University of Alagoas, Maceió, Alagoas Brazil; 3 Department of Public Health, Federal University of SC, Florianópolis, Santa Catarina Brazil; 4 Department of Food, Nutrition, and Packaging Sciences, Clemson University, Clemson, South Carolina USA

**Keywords:** University students, Cooking classes, Cooking intervention, Culinary skills, Real setting, Cooking behavior, Healthy feeding practices, Sustained impact, Methodology, Study protocol

## Abstract

**Background:**

Community-based intervention studies that aim at developing cooking skills have increased in the scientific literature and are related to healthier food practices. However, methodological limitations are observed and only a few studies have university students as the target. The university entrance period has been related to negative changes in eating habits among young people and it represents an important period for developing interventions for health promotion. This study describes the study protocol and the evaluation framework for the Nutrition and Culinary in the Kitchen program. This program aims to develop cooking skills in university students, and is based on the Cooking with a Chef program in the United States.

**Methods:**

This ongoing, randomized controlled intervention was designed with a six month follow-up study. The intervention consisted of three-hour weekly classes during a six week period with printed materials provided. Five of the classes were hands-on cooking and one was a tour to a popular food market. There were eight primary outcome measures: changes in relation to i) accessibility and availability of fruits and vegetables; ii) cooking attitudes; iii) cooking behaviors at home; iv) cooking behaviors away from home; v) produce consumption self-efficacy; vi) self-efficacy for using basic cooking techniques; vii) self-efficacy for using fruits, vegetables, and seasonings (while cooking); and viii) knowledge of cooking terms and techniques. Secondary outcomes included changes in body mass index and in personal characteristics related to cooking. Repeated measures were collected through the application of an online self-completed survey, at baseline, after intervention and six months after intervention. A sample of 80 university students (40: intervention group; 40: control group) was estimated to detect a mean change of 1.5 points in cooking knowledge, with study power of 80%, and 95% level of confidence, plus 20% for random losses and 10% for confounding factors. The control group participants have continued with their usual activities. Data analyses will evaluate the intervention effect on changes in outcomes within and between groups, as well as explore relations with personal characteristics.

**Discussion:**

This method provides new evidence about whether or not a culinary intervention targeting university students has an impact on the improvement of cooking skills and healthy eating practices.

**Trial registration:**

Brazilian Clinical Trials Registry - RBR-8nwxh5 (http://www.ensaiosclinicos.gov.br/rg/RBR-8nwxh5/)

## Background

Studies have demonstrated that, when entering the university some students present with inadequate food habits. These habits are characterized by the increased consumption of snacks, fast food, French fries, sweets, cakes and pies, soft drinks, and the reduced consumption of fruits and vegetables [1–4]. In addition, the first years at the university are associated with weight gain and increase in the prevalence of overweight and obesity, also related to the potential increased risk of chronic diseases [5–8]. However, there is contrasting literature that suggests weight gain may be related to other factors such as socioeconomic status and social responsibilities, rather than university attendance. It is noted that college students and non-college individuals gain similar amounts of weight at comparable ages [9].

University students have related some barriers that inhibit their adoption of healthier food habits, such as lack of time, money and knowledge about cooking skills and how to prepare their own food; lack of space and kitchen utensils and equipment; living away from parents home; and availability and access to unhealthy and convenience foods [2, 10–16].

Studies have discussed that changes in how to prepare and cook foods can influence individuals’ cooking skills and may be related to the transfer of cooking knowledge between parents and their children as well as within the school setting [17–19]. Such changes may be also related to the possible restructuring in the mode of preparing food at home, making use of technology (such as the microwave oven) and of ready-to-eat food products to facilitate meal the preparation [17]. These social trends in time use, transfer of cooking skills and food purchasing can influence the time one spends in the kitchen. Lyon et al. (2011) analyzed food practices by younger and older women in Scotland and identified that differences in these practices are related to current lifestyle factors. Thus, in this study, women had different levels of cooking knowledge, but they shared similarities in food practices in the kitchen [20]. At the same time, there has been an increase of studies in the scientific literature about cooking skills as related to healthier eating habits [21–24].

In this respect, studies have reinforced the importance of encouraging intervention programs that aim to develop cooking skills [22, 23, 25–27], by means of changes in cooking knowledge, attitude, and behavior related to healthier eating habits [23, 28].

Reicks et al. [23] added the topic of the health impact of home cooking on adults to the literature. The main outcomes measured were dietary intake, knowledge or skills, cooking attitudes and self-efficacy, and health outcomes. Among the studies in this review set, only half had a control group and the follow-up period varied from one to forty-eight months. In this context, the authors highlight the broad methodological variability of studies, including the lack of methodological rigor, as well as the use of non-validated instruments to evaluate cooking interventions. Therefore, the authors reinforce the need to evaluate such interventions in the long term, so that there may be consistent evidence to relate cooking skills with outcomes in nutrition and health [21, 23, 29].

In relation to cooking interventions involving university students, four studies were found in the scientific literature [30–33], albeit only one of them used a validated instrument for this target population [32]. This instrument was developed in the United States (U.S.) at Clemson University, and consists of the measurement of cooking skills related to health and nutrition to evaluate the intervention program Cooking with a Chef (CWC).

It is relevant to note that interventions using a validated instrument with a focus on cooking skills that evaluate the sustained impact [34, 35] on the participants’ eating practices have not yet been found in Brazil. In addition, the current food guide for the Brazilian population highlights, in one of the guidelines, the importance of developing, practicing, and sharing cooking skills as well as valuing the art of preparing and cooking food for the promotion of healthy eating [36].

Thus, the literature converges to emphasize the importance of implementing cooking interventions with university students that aim at evaluating the sustained effect on the development of healthier eating practices. For this purpose, we stress the need for adapting the existing CWC intervention program and its evaluation instrument for the culture of the place where it is intended to be used. The purpose of this methods paper is to describe the study protocol and the evaluation framework of the intervention program Nutrition and Culinary in the Kitchen (NCK), designed for Brazilian students, based on the U.S. program CWC.

### Nutrition and culinary in the kitchen program, Brazil

The NCK program was designed based on the U.S. CWC program, that, in turn, was extensively applied with different target populations in the U.S. [32, 37–43]. A questionnaire was developed for the CWC program and presents predictive and construct validity [44]. This instrument contains evaluation scales about fruit and vegetable consumption as well as about different dimensions of cooking skills, including cooking knowledge, cooking behaviors and cooking attitudes.

Condrasky [28] has worked with the development and evaluation of programs and interventions that focus on nutrition and culinary concepts since the 2000’s. The CWC program involves hands-on cooking classes, conducted by a nutritionist and a chef, while the NKC program is conducted by a dietitian with experience in dietetics and cooking techniques. Besides, the program works with basic cooking techniques as well as easy to comprehend nutritional information of food for people with limited experience in the kitchen [32, 37–42].

The CWC program was adapted specifically to the Brazilian population, generating the NCK program. Such adaptation was made during a period of eight months and followed the stages:

1. Completion of a five-month internship by the main researcher (G.L.B) to follow the original CWC program on-site at Clemson University, U.S.;
2. Definition of guiding principles based on national and international guidelines for the promotion of healthy eating to adapt the CWC program to Brazil;
3. Implementation of consensus workshops with experts to define the modifications for the program in Brazil;
4. Development, adaptation, and testing of 32 recipes to be used in the adapted program;
5. Evaluation of the adequacy of the recipes in relation to sensory characteristics (color, odor, appearance, texture, and flavor) and ultimately applying these sensory criteria to recipes deemed healthy; and
6. Pilot testing of the program as a cooking class with a similar target population of University students.

Taking into account the cultural differences between the United States and Brazil, ten guiding basic principles were developed for the adaptation of the CWC program to Brazil. These principles were created based on several public policies on healthy eating proposed by the World Health Organization [45–48], on the food guides for the Brazilian population proposed by the Ministry of Health [36, 49], as well as on the experiences of the researchers and the research group (standardization of healthy menus, control of trans fat and sodium in preparations, analysis of labels of industrialized food products, among others) [50–59] (Table 1).

**Table 1:** The 10 basic principles for the adaptation of Cooking with a Chef program within the Brazilian Nutrition and Culinary in the Kitchen program

Basic principles	
1	Appreciation of food in natural form or minimally processed foods, preferably organic and from agro-ecological agriculture, respecting seasonality.
2	Importance of a healthier menu planning considering the grocery list development, pantry organization, and meal preparation.
3	Planning healthier meals based on food groups and subgroups and portion size recommendations. Encouraging the use of fruits, vegetables, whole grains, and nuts.
4	Enhancement and maintenance of nutritional and sensorial quality during the food preparation process.
5	Knowledge and practice of healthier cooking techniques, considering techniques of food pre-preparation, preparation and distribution.
6	Limitation in the use of processed foods and elimination of ultra-processed foods.
7	Elimination of ingredients with industrial trans fatty acids in culinary preparations.
8	Decreased salt use for preparations and encouragement for the use of fresh herbs, spices and condiments that are minimally processed.
9	Limitation in the use of ingredients containing free sugars, added sugars or artificial sweeteners.
10	Understanding food nutritional information, enabling reading and analysis of the labels prior to purchase with respect to the amount per serving and serving size, the ingredient list and the nutrition facts label.

Notes: Based on references [36, 45–59]

#### Objectives of the NCK program, Brazil

The Nutrition and Culinary in the Kitchen (NCK) program was developed to transfer knowledge about nutrition science and culinary techniques. The program allows for participants to practice cooking skills so that they are able to feel comfortable and confident enough to prepare healthier food and to make nutritious ingredient choices. The program promotes healthier food habits by means of hands-on cooking classes based on the food groups, menu planning, basic cooking techniques, tips for optimizing productivity in the kitchen, as well as skills to prepare meals.

#### Theoretical perspectives

Intervention studies that aim to promote healthy eating habits should have a solid theoretical foundation designed for enhancing knowledge and positively influencing health behavior [60]. The CWC and NCK programs were based on the Social Cognitive Theory (SCT) proposed by Bandura. The SCT model involves interpersonal and environmental influencing factors as related to behavior. SCT helps to focus on the analysis of reciprocal interactions among people and environment as related to behavior [61, 62].

Self-efficacy is a central construct in SCT used to determine change in behavior. It refers to the confidence to overcome obstacles and successfully achieve a particular behavior [60, 62]. Culinary skill self-efficacy that are measurable may be effective at identifying positive changes in such behaviors [44, 63]. It is necessary, however to offer opportunities to practice such learned behavior as well as to provide for positive reinforcement in order for learning to take place [62]. Practice is important to encourage confidence once participants prepare part of a meal for the group using information and skills learned during the program [63]. As identified within SCT, behavior can be changed through new learning experiences, guidance in the adjustment of perceptions, and through support for the development of capacities. [60]. Within NCK classes, participants cooking behaviors are practiced such as: knife skills of slicing, dicing and cutting, basic cooking techniques (i.e. roasting, sautéing, and pressure cooking), food preservation techniques (i.e. blanching), and nutrition labeling analysis to facilitate healthy food choices. Also, in the NCK program, positive reinforcement takes place during the hands-on cooking classes by means of verbal comments made by the dietitian that conducts the program throughout the planning and execution phases of food preparations. In addition, at the end of each class, the moment called “Seasoning ideas” takes place, during which there is a discussion about the themes and key points covered in the class. These discussions employ a structured script with questions. The objective is to encourage the exchange of positive experiences of that particular class in relation to cooking and nutrition among the participants.

## Methods/design

### Study design

This study used a randomized controlled trial design with six months of follow-up (with repeated measures) to test a community-based cooking skills program to improve cooking and healthy eating behaviors in university students. The study started in 2015 with follow-up studies planned for 2016 and 2017.

The intervention program occurred over a two-month period, one day a week for eight weeks, with two weeks having no intervention topics (due to holiday schedule). The intervention group (IG) data was analyzed at three-time points: (1) on the week prior to the program’s beginning (T1); (2) on the completion of the eight-weeks program (T2); and (3) after six months, on the intervention follow-up period (T3). The control group (CG) comprised participants from the wait-list group who were waiting for 12 months to participate in the study as they continued with their usual practices. Data from this group was analyzed at the same three-time points before they began participation in the program and subsequent comparison with the IG data. After that, the CG will be invited to take part of the NCK program in order to be able to receive the program benefits. Once the study is ongoing, the control group will be invited to participate in the NCK program at the next stage of the study. Thus, the impacts of the intervention program will be evaluated immediately following the post intervention (T2) and the sustained effect, six months after the program (T3) [34, 35] for both groups (CG & IG).

### Study population

The target population of the study was composed of regularly enrolled university students who began their first year of an onsite undergraduate course in a public Brazilian university. A representative sample of these students, who were 16 years old or older, participated in the validation stage of the evaluation instrument on cooking skills and healthy eating practices. Excluding criteria involved: students enrolled in graduate courses or distance education undergraduate courses, and students who were enrolled in or after the second year of an onsite undergraduate course. A minimum of 770 participants was necessary for this stage of the study, considering possible attrition of 10% by follow-up stage, 2.0 effect sample size and 5% of random error. In total, 767 university students were considered eligible to participate in the validation stage of the instrument.

### Sample size calculation

Sample size calculation for the intervention aimed at detecting changes in the average values of the outcome related to cooking skills knowledge [32]. Considering a difference of 1.5 points in average [32], with study power of 80%, an error rate of 5% and a 95% level of confidence, a sampling plan of 28 students was estimated. Including a random loss of 20% and 10% for possible confounding factors, a minimum sample of 40 students was suggested to be investigated in each group (intervention & control), involving a total sample of 80 participants. Sample size calculation was carried out with the statistical program Open Epi version 3.03 (Open Source Epidemiologic Statistics for Public Health, Atlanta, GA, USA).

### Sample recruitment and selection

Sample recruitment and selection of participants for the culinary intervention were carried out taking into consideration some inclusion criteria: (1) being 16 years old or older; (2) having participated in the validation stage of the evaluation instrument on cooking skills and healthy eating practices; (3) not living with parents; (4) having a kitchen with basic equipment and utensils available (stove or microwave oven, refrigerator, cutlery, and pans) to prepare their food; (5) demonstrating interest and availability in participating in the cooking classes; and (6) signing the Consent Form. Students that participated in the validation stage of the instrument and met the inclusion criteria were eligible to participate in the intervention. Lead researcher of this study sent, an electronic (e-mail) invitation to participate to the students that fit the criteria for inclusion.

**Fig. 1:**
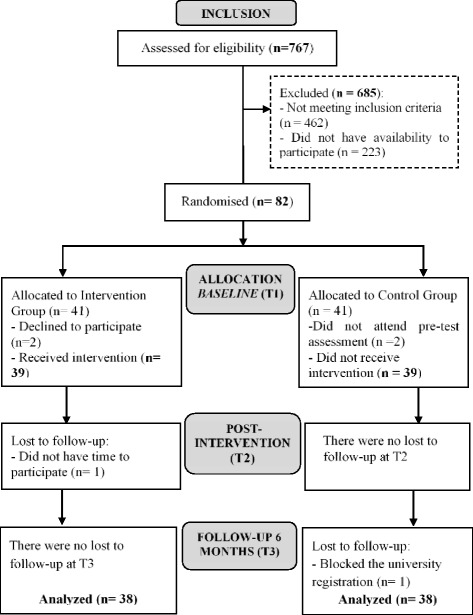
Flow diagram of participant recruitment during the trial according to CONSORT

In total, 305 students were considered eligible. From this number, 82 accepted invitation to participate in the intervention: 41 in the IG, and 41 in the CG. The diagram of participant recruitment is presented in Fig. 1.

### Randomization

Participants who were accepted to take part in the study were randomly assigned to groups (IG & CG), to ensure homogenization and a similar number of participants in each of the groups. Participants’ random order to each group was determined with the help of the online software *Research Randomizer* (https://www.randomizer.org/). Students were not informed about which group they were assigned since all students would have the opportunity to participate in the NCK program in time. Control group participants were informed that they would take part in the intervention program after answering online surveys in three distinct moments (T1, T2, and T3), characterizing them as part of the wait-list control group.

### Intervention

The NCK program was structured into five hands-on cooking classes and a food selection and purchase workshop to a popular food market. Three hour classes for these six sessions provide a total of 18 h of intervention. The classes took place in the food laboratory, which resembled a home kitchen with utensils and home style equipment. This setting provided ease of access for students to work rather than them using an industrial kitchen laboratory lay out. Each hands-on cooking class presented specific objectives, and in the course of each class, the topics learned in previous classes were reinforced by means of practice in the kitchen. All cooking classes were characterized as hands-on and included different demonstrations of ingredients and cooking techniques; discussions about nutrition; preparation of recipes by the participants; as well as eating the meal and discussion at the end of each class session. Each class was conducted with a group of 10 to 12 students, divided into groups of two to three students per workbench, enabling all students to practice what was demonstrated in the class. The selection and purchase workshop involved different dynamics. Students were taken to a popular food market where fresh and raw food products were available for purchase, as well as fruits, vegetables, fish, meats and breads. This range of products and local foods was estimated to be at affordable prices for most students.

#### Development and adaptation of recipes to the NCK program, Brazil

Taking into consideration the guiding principles for the adaptation of the CWC program for Brazil, recipes of the NCK program were developed and included. To reach such a goal, the following aspects were considered: (1) number of hands-on cooking classes to be administered; (2) number of recipes to be worked within each class; (4) target population; (5) average time to prepare recipes, to provide greater flexibility for culinary preparation; (6) utensils and ingredients to prepare the recipes; (7) average cost of each culinary preparation; (8) culinary techniques to be taught in each class; and (9) criteria to consider the recipe as healthy.

The criteria to consider a recipe as healthy were based on national and international public policies in the context of healthy eating, such as: (1) preference for food in its natural form (fresh) and minimally processed foods; (2) limited use of salt and sugar; (3) use of herbs and spices; (4) use of preparation techniques that are considered healthier (e.g.: baking, roasting, sautéing, pressure cooking); (5) use of vegetables respecting seasonality; and (6) elimination of ultra-processed foods as well as of products with trans fatty acids ingredients [36, 45–49]. Because NCK recipes were used by university students, additionally preparation time, level of difficulty and the cost of ingredients were considered. These criteria have been cited by this population as possible barriers for preparing meals at home [10, 14–16]. Each of the recipes was cooked from scratch, including preparations based on fruits and vegetables within each of the cooking classes. Most of the ingredients were in their natural (fresh) form or in minimally processed foods. In addition, the feasibility of employing the recipes from the CWC program was verified using the decision tree model. All the recipes were tested and evaluated using a standardized form. Recipe evaluation criteria involved sensory characteristics (appearance, color, odor, texture and flavor), as well as the criteria list used to consider a recipe as healthy.

Table 2 describes the weekly planning of the NCK program and the recipes prepared by the participants in each hands-on cooking class.

**Table 2:** Weekly planning of the Nutrition and Culinary in the Kitchen program including the recipes prepared by the participants in each hands-on cooking class

Week	Classes	Aim	Recipes	Cooking Techniques
1	Cooking class 1	Learning basic cooking techniques and cooking skills to prepare a pleasurable, healthy and easy meal	- Roasted vegetables - Fresh fruit salad - Omelet - Sautéed, baked and pressure chicken^a^ - Homemade broth of vegetables and chicken	- Roasting - Stir-frying - Sautéing - Pressure cooking
2	Cooking class 2	Getting to know the importance of including more fruits and vegetables in the diet daily	- Baked chicken and vegetable Salad - Homemade yogurt sauce with parsley - Fresh fruit cream sherbet - Whole-meal bread made in the frying pan	- Roasting - Blanching - Grilling
3	Selection and purchase workshop	Getting to know a place where fruits, vegetables, meats, and fish are sold, as well as learning how to choose foods and to understand food nutrition labeling	- There is no preparation of recipes in this class - Participants will have the opportunity to get to know and to buy fresh foods, especially fruits and vegetables at affordable prices	
4	Cooking class 3	Learning how to produce a healthy and complete meal from food products available in the pantry	- Whole-grain rice with garlic - Black beans cooked with pumpkin - Brazilian style beefsteak with onions - Mixed salad - Homemade vinaigrette sauce - Fresh orange	- Blanching - Grilling - Stir-frying - Pressure cooking
5	Cooking class 4	Learning the importance of consuming whole grains and of considering flavor during meal planning	- Roasted homemade meatballs - Whole-wheat pasta - Homemade tomato sauce - Broccoli salad with lentils - Homemade salad dressing - Fresh Fruit platter	- Roasting - Blanching - Boiling - Stir-frying
6	Cooking class 5	Using the cooking skills practiced in all the classes to produce a complete meal	- Parboiled rice with parsley - Stewed fish with coconut milk (*Moqueca*) - White bean salad with onions, lettuce and tomatoes - Orange, mustard and honey salad dressing - *Farofa* ^a,b^ - Fish *Pirão* ^a,c^ - Whole-meal cake	- Baking - Blanching - Stewing - Boiling - Stir-frying - Pressure cooking

Notes: ^a^Culinary preparations executed by the guide who was responsible for the class with the objective of teaching, by means of demonstrations, a new cooking or preparation technique;

^b^ *Farofa*: dish of Brazilian kitchen made of manioc flour fried in fat (oil or butter), which can be enriched with other ingredients (vegetables, egg, meats);

^c^ *Pirão*: dish of Brazilian kitchen made of manioc flour cooked in a hot stock (broth)

#### Intervention stages

Table 3 describes the stages for the cooking intervention as well as the procedures for the intervention and control groups.

**Table 3:** Evaluation measures for university students participating in the Nutrition and Culinary in the Kitchen program

Meeting	Evaluation measures	Intervention group	Control group
1	Online survey at baseline (T1)	Cooking class 1	E-mail contact with a link to the online survey
2	No data collection	Cooking class 2	No intervention
3		Selection and purchase Workshop	
4		Cooking class 3	
5		Cooking class 4	
6	Online survey to be completed right after intervention (T2)	Cooking class 5	E-mail contact with a link to the online survey
7	Follow-up: online survey to be completed six months after intervention (T3)	E-mail contact to answer the survey online	E-mail contact with a link to the online survey
8	–	–	E-mail contact with an invitation to participate in the intervention program

Nutrition undergraduate and graduate students were invited to participate in the facilitators’ group to help during the hands-on cooking classes. They received a theoretical-practical training of 3.5 h that included an explanation on the presentation of each hands-on cooking class, as well as a guided tour to the lab kitchen where the classes would take place. In each hands-on cooking class, there were from four to five facilitators and a coordinating teacher for each group of 10 to 12 students. The coordinating teacher had an undergraduate degree in Nutrition and practical experience in culinary. Though there are a large number of facilitators to assist in the hands-on cooking classes, given guidance from SCT this level of guidance (social support) was considered to be important. Guidance from facilitators encouraged participants culinary skills efforts and contributed through guided practice to attempt to enhance self-efficacy [60].

A pilot study of the first hands-on cooking class was carried out with a group of ten students who were enrolled in their first year of an undergraduate course of the same university to identify any possible adjustments in the presentation of the classes, as well as in the educational material to be used. Materials were adapted from the CWC program and included the following topics that were developed to each cooking class: i) an overview of the cooking class, including its objectives; ii) class agenda, with suggestions on the distribution of time for each activity; iii) instructions about the organization of material and participants for the cooking classes; iv) conceptual issues about nutrition and cooking in relation to the class theme; v) complete recipes as demonstrated by the coordinating teacher and prepared by the participants; and vi) issues for discussion, reflection and challenges subsequent classes (“Seasoning ideas” moment).

### Outcomes measures

#### Primary outcomes

i) accessibility and availability of fruits and vegetables; ii) cooking attitudes; iii) cooking behaviors at home; iv) cooking behaviors away from home; v) produce consumption self-efficacy; vi) self-efficacy for using basic cooking techniques; vii) self-efficacy for using fruits, vegetables, and seasonings (while cooking); and viii) knowledge of cooking terms and techniques; assessed at baseline, immediately after intervention, and six months after intervention.

#### Secondary outcomes

Body mass index (BMI) of university students as well as their socio demographics and characteristics related to cooking; assessed at baseline, immediately after intervention, and six months after intervention.

### Survey instrument

The evaluation instrument of cooking skills and healthy eating employed in the present study was developed for the CWC program and validated by Michaud [63]. This questionnaire was originally applied in a written form to parents, caregivers [40], and cooks [41]. An online form was given to university students [32]. The NCK instrument was self-administered in online form with use of tablet computers. The use of tablet computers allowed for time optimization and facilitated the survey self-completion by students during the classes. The instrument was cross-culturally adapted to Brazil, according to the following procedures: 1) translation (two independent translations); 2) synthesis of translations (discussion among two translators and one researcher); 3) back-translations (two independent translations compared with the original questionnaire, followed by a discussion among dietitians); 4) expert committee (consensus workshop with experts and university students); 5) synthesis of the final Brazilian Portuguese version; and 6) pre-test (self-administered online questionnaire by 48 university students) [64, 65]. Original and translated questionnaires were compared for conceptual, item, semantic (idiomatic and experiential), and operational equivalencies [66].

The questionnaire items were distributed within eight measures about cooking skills and healthy eating, and a group of items about personal, cooking and demographic characteristics. The process of cross-culturally adaptation and validation of the instrument to Brazil is ongoing and the first paper was published in a scientific journal [67].

Participants were asked to report their demographics and personal characteristics including: age, gender, undergraduate course, type of university admission, parental education level, ethnicity, ascendance, if they have children <16 years old, as well as with whom they live. Cooking characteristics such as daily time available to cook, equipment and utensils available at home, self-reported cooking knowledge, source of cooking experience and lunch or dinner location were also collected to characterize the population of the study. Height and weight were self-reported to enable the calculation of BMI. Table 4 provides a description of each evaluation measure listing instrument or process used and type of data collected by each measure.

**Table 4:** Evaluation measures for university students participating in the Nutrition and Culinary in the Kitchen program^a^

Target measurement	Instrument/Process	Description
Demographic characteristics	Student self-report	Birthdate, gender, ethnicity, ascendance, parental education level; composed of six items^b^.
Personal characteristics	Student self-report	Undergraduate course, type of university admission, if they have children <16 years old, with whom they live; composed of four items^b^.
Cooking characteristics	Student self-report	Daily time available to cook, equipment and utensils available at home (39 items), self-reported cooking knowledge, source of cooking experience; and lunch or dinner location; composed of six items^b^.
Height and weight	Student self-report	Student self-report as part of the online survey to enable the calculation of BMI^b^.
Accessibility and Availability of Fruits and Vegetables Index (AAFV)	Cooking skills and health eating questionnaire	Availability of fruits and vegetables over the previous week; composed of eight items with yes/no questions, scored as 1 or 2, respectively^b^.
Cooking Attitude (CA)	Cooking skills and health eating questionnaire	How respondents felt about cooking; composed of seven items with 5-point Likert scale responses (from “strongly disagree” to “strongly agree”) ^b^.
Cooking Behavior at home (CBH)	Cooking skills and healthy eating questionnaire	Frequency of common cooking activities at home; composed of six items with 5-point Likert scale responses (“not at all”, “1 to 2 times a month”, “once a week”, “several times a week”, and “about every day”)^b^.
Cooking Behavior away from home (CBAH)	Cooking skills and healthy eating questionnaire	Frequency of common cooking activities away from home; composed of five items with 5-point Likert scale responses (“not at all”, “1 to 2 times a month”, “once a week”, “several times a week”, and “about every day”)^b^.
Produce Consumption Self-Efficacy (SEPC)	Cooking skills and healthy eating questionnaire	Degree of confidence in meeting the government’s recommendations for the consumption of fruits and vegetables; composed of three items with 5-point Likert scale responses (from “not confident at all” to “extremely confident”)^b^.
Self-Efficacy for Using Basic Cooking Techniques (SECT)	Cooking skills and healthy eating questionnaire	Degree of confidence in performing basic cooking techniques; composed of 18 items with 5-point Likert scale responses (from “not confident at all” to “extremely confident”) ^b^.
Self-Efficacy for Using Fruits, Vegetables, and Seasonings (while cooking) (SEFV)	Cooking skills and healthy eating questionnaire	Degree of confidence in using fruits and vegetables when cooking; composed of nine items with 5-point Likert scale responses (from “not confident at all” to “extremely confident”) ^b^.
Knowledge of Cooking Terms and Techniques (CCT)	Cooking skills and health eating questionnaire	Level of cooking knowledge; composed of eight items with multiple choice answers (correct answer scored as 1 point) ^b^

^a^Measures will be collected at baseline (week 1), immediately post intervention (week 6) and 6 months after the end of intervention (follow-up)

^b^Student self-report in the online survey

### Statistical analysis

Descriptive analysis of the sociodemographic variables and of the student characteristics at baseline will be conducted with measures as mean and standard deviation for symmetrical numeric variables, or median and interquartile interval for asymmetrical numeric variables. For categorical variables, the description involves the prevalence of data (absolute and relative frequencies).

T-test (for parametric data) or Mann-Whitney test (for non-parametric data) will be run to verify the differences intragroup, among control groups and intervention for the pre-intervention condition (baseline) with continuous variables. For categorical and parametric variables, chi-square test or chi-square fisher’s exact test will be used.

Intervention’s effect will be evaluated by means of the analysis on the changes in cooking skills and healthy eating survey. Issues related to the scales evaluated by the instrument (in T1, T2, and T3) will be analyzed to verify data normality. Subsequently, ANOVA for repeated measures (for parametric data) or Friedman (for non-parametric data) followed by Wilcoxon signed rank test (post-hoc test) will be employed.

Data collected through the online survey will be transferred to Microsoft Office Excel® and exported to Stata® version 13.0 (Statacorp, College Station, TX, USA). Statistical analysis will be run on Stata® and a significance level of 5% will be adopted, considering *p* < 0.05.

## Discussion

The evaluation of the NCK program may contribute to the growth of scientific literature in relation to the effectiveness of community-based culinary interventions. The randomized controlled methodological design with a follow-up after intervention will allow the researchers to analyze the sustained effect of the program on the eating practices of the chosen population. In addition, the methodological design described in this paper will permit the comparison of results with other culinary intervention programs conducted with similar populations. Since it is the first culinary intervention study conducted in Brazil that is controlled and randomized, as well as the first sustained impact study with university students at an international level, the study may contribute to the generation of scientific knowledge to develop public policies that aim at promoting healthy eating by means of the practice of cooking skills with this population.

This paper also provides practical information about the design and presentation of community-based interventions, including clear details about the program and the recruitment of the intervention and control groups. The use of recipes that were developed, adapted and tested for the objectives of the NCK program and to the target population of this study may contribute to the retention of participants in the program, as well as to the development of cooking skills and healthier eating habits.

The present study also presents some limitations. The population of the study belongs to only one public university, a fact that does not allow for the generalization of results. However, the chosen university has more than 30,000 enrolled students in undergraduate courses. Students come from several regions of Brazil and South American countries, which may contribute an increased diversity within the study sample. The inclusion of students from the same university cannot guarantee that the control group will not be influenced by the intervention group. When the control group waits until the study concludes to receive the intervention it can present limitations. During the waiting period, they may have experiences that may impact study outcomes [68]. Nevertheless, randomization procedures within the study groups remain the only method that could eliminate possible selection and confounding biases [69]. The chosen university has faculty, employee, and student population of 50,000 who circulate every day on campus. Thus, the likelihood of students meeting and influencing counterparts on culinary skills may be diminished. As such this factor presents a limitation on community-based intervention studies in general.

Additionally, the online survey was composed of questions that were answered by the participants themselves, which may allow for some information bias. For the BMI classification, data about weight and height provided by the participants themselves were used, which likewise may allow bias in regards to underestimation of weight or overestimation of height. Such bias may occur with overweight participants who tend to underestimate their weight [70]. However, self-reported measures of weight and height have been considered valid in epidemiologic studies and may be used to improve accuracy of the collected data [71].

Given that the study involves a six month follow-up, it is prone to have subject losses during the follow-up period. However, contact with participants will be kept through cell phone electronic messages to remind and reinforce participant attendance at follow-up encounters. Thus, attrition will be recorded in order to verify whether there are differences among students that remained in the program.

One of the strengths of the present study is conducting the intervention by means of a hands-on cooking class series, as opposed to providing a series of cooking demonstration classes. While there is a high staff ratio to assist the participants, the study team assumed that this approach is important for the development of hands-on cooking skills. As a strategy for future program implementation the team would consider a train the trainer model with supervision for interested students. By expanding the trainer pool at a university or like setting the replicability of hands-on cooking classes may be manageable. Studies have suggested that changes in behaviors, attitudes and knowledge about cooking are more substantial among those who have had hands-on cooking classes when compared to those who only participated in expository lectures or classes with a specific interface, as a television [29–31, 72].

NCK class encounters take place in a real home kitchen type setting. Thus, these characteristics may allow for the application of the program in other university types of environments. The instrument employed in the original CWC program was validated for the population of the study that, in turn, was culturally adapted and validated for Brazil [67]. Taking into consideration that few culinary intervention studies use validated and/or adapted instruments with the target population [23], the design of the present study follows methodological rigor.

Furthermore, this study made use of an instrument that was developed specifically for the intervention program. This instrumentation included components to evaluate healthy eating and the cooking practices that were practiced during the hands-on cooking classes, such as, the blanching technique.

It is well known that sustained impact studies of culinary intervention with adults are scarce in the literature [29, 34, 35]. The results of this study, which uses a randomized, control design and with a six month follow-up culinary intervention model will allow high quality evidence to evaluate the impact of the NCK program, based on the CWC program, on participants’ eating habits and cooking behavior. This is the beginning of a program that aims at reaching the entire university community. It is the first cooking skills intervention study devoted to the university community in Brazil. The program may also contribute to the scarce evidence in the literature about the effectiveness of culinary intervention studies with a follow-up period in adults.

## Abbreviations

AAFV: Accessibility and Availability of Fruits and Vegetables Index
BMI: body mass index
CA: Cooking Attitude
CBAH: Cooking Behavior away from home
CBH: Cooking Behavior at home
CCT: Knowledge of Cooking Terms and Techniques
CG: Control group
CWC: Cooking with a Chef
IG: Intervention group
NCK: Nutrition and Culinary in the Kitchen
SCT: Social Cognitive Theory
SECT: Self-Efficacy for Using Basic Cooking Techniques
SEPC: Produce Consumption Self-Efficacy
SEVS: Self-Efficacy for Using Fruits, Vegetables, and Seasonings (while cooking)
